# Apport du Holter ECG dans le bilan étiologique des infarctus cérébraux à Brazzaville, Congo

**DOI:** 10.11604/pamj.2018.31.235.17709

**Published:** 2018-12-19

**Authors:** Stéphane Méo Ikama, Jospin Makani, Ghislain Mpandzou, Paul Macaire Ossou-Nguiet, Bernice Mesmer Nsitou, Munka Nkalla Lambi, Edgard Matali, Thierry Raoul Gombet, Suzy Gisèle Kimbally Kaky

**Affiliations:** 1 Service de Cardiologie, Centre Hospitalier Universitaire de Brazzaville, Congo; 2 Service de Neurologie, Centre Hospitalier Universitaire de Brazzaville, Congo

**Keywords:** Infarctus cérébral, Holter ECG, fibrillation atriale, Congo, Ischemic stroke, Holter ECG, atrial fibrillation, Congo

## Abstract

Déterminer la prévalence des troubles rythmiques au cours des infarctus cérébraux et identifier les facteurs prédictifs de la fibrillation atriale (FA) paroxystique. Il s'est agi d'une étude transversale, descriptive et analytique, menée à Brazzaville entre janvier 2012 et décembre 2016. Elle a porté sur une série consécutive de 267 patients victimes d'un accident vasculaire cérébral ischémique transitoire (n = 17) ou constitué (n = 250), documenté par un scanner cérébral. Tous ces patients ont bénéficié d'un enregistrement Holter ECG dès 24h, réalisé dans le cadre de la recherche étiologique. Les principales anomalies rythmiques enregistrées ont été répertoriées et la régression logistique a permis l'identification des facteurs prédictifs de survenue de la FA paroxystique. Il s'agissait de 164 hommes (61,4%) et 103 femmes (38,6%), âgés en moyenne de 60,2 ± 12,1 ans (extrêmes: 22 et 94 ans). Les principaux facteurs de risque cardiovasculaire identifiés étaient une hypertension artérielle (HTA) dans 214 cas (80,1%), un diabète sucré dans 36 cas (13,5%), et un tabagisme dans 18 cas (6,7%), avec un taux de cumul de 1,5 facteur par individu. L'examen Holter ECG, normal dans 216 cas (81%), était pathologique dans 51 cas (19%). Les principales anomalies enregistrées consistaient en des extrasystoles ventriculaires bénignes (n = 32), une FA paroxystique (n = 7), des extrasystoles supraventriculaires (n = 5), une tachycardie ventriculaire (TV) non soutenue (n = 4), une TV soutenue (n = 2) et un bloc auriculo-ventriculaire type Mobitz II (n = 1). La fréquence de la FA paroxystique était de 2,6%. En analyse bivariée, il n'a pas été noté de corrélation entre la FA paroxystique et le sexe (p = 0,890), l'HTA (p = 0,818), le diabète (p = 0,839), le tabac (p = 0,969). En analyse multivariée, seul l'âge était prédictif de la survenue d'une FA paroxystique au cours des infarctus cérébraux (OR = 1,11;p = 0,0134). Il ressort de cette étude préliminaire que les troubles du rythme emboligènes sont relativement rares au cours des infarctus cérébraux à Brazzaville. La FA paroxystique, quoique peu fréquente, reste essentiellement corrélée à l'âge. Sa recherche systématique chez les sujets âgés contribue à améliorer la prise en charge.

## Abstract (en)

To determine the prevalence of the rhythmic disorders during ischemic stroke, and to identify the predictive factors of paroxysmal atrial fibrillation (PAF). It was about a cross-sectional study, descriptive and analytical, conducted to Brazzaville between january 2012 and december 2016. It related to a consecutive series of 267 patients victims of a transient ischemic attack (n = 17) or ischemic stroke (n = 250), documented by cerebral tomodensitometry or brain MRI. All these patients profited from a recording 24h Holter ECG, carried out within the framework of etiologic research. The principal recorded rhythmic anomalies were indexed and the logistic regression allowed the identification of the predictive factors of PAF. They were 164 men (61.4%) and 103 women (38.6%), old on average of 60.2 ± 12.1 years. The identified cardiovascular risk factors were hypertension (80.1%), diabetes (13.5%), and tobacco use (6.7%). The 24h Holter ECG, normal in 216 cases (81%), was pathological in 51 cases (19%). The principal recorded anomalies consisted into ventricular ectopic beats (n = 32), PAF (n = 7), supraventricular ectopic beats (n = 5), non-sustained ventricular tachycardia (n = 4), sustained ventricular tachycardia (n = 2), and type 2 atrio-ventricular block (n = 1). The frequency of PAF was 2.6%. In bivariate analysis, it was not noted correlation between PAF and sex (p = 0.55), hypertension (p = 0.42), diabetes (p = 0.64), and tobacco use (p = 0.61). In multivariate analysis, only the age was the predictive factor of PAF during ischemic stroke (p = 0.0134). It comes out from this preliminary study that the emboligenous arrhythmias are relatively rare during ischemic stroke in Brazzaville. PAF, though little attends, remains primarily correlated at the age. Its systematic research at the old subjects contributes to improve the assumption of responsibility.

## Introduction

L'ischémie cérébrale constitue un problème majeur de santé publique dans le monde. En effet, l'incidence des maladies cardiovasculaires, en particulier l'infarctus du myocarde (IDM) et les accidents vasculaires cérébraux (AVC), est d'environ 32 millions de cas par an dans le monde, dont 12,5 M d'accidents fatals [1-4]. D'après les projections de l'OMS d'ici à 2020, les maladies cardiovasculaires seront responsables de 25 millions de décès dans le monde, dont 19 millions dans les pays en voie de développement [5]. L'ischémie cérébrale est à l'origine de 30% de décès dans les trois premières semaines de survenue d'un AVC et responsable de 30% de handicap physique permanent [5]. Les embolies et l'athérothrombose des gros vaisseaux constituent les principaux mécanismes étiologiques de l'ischémie cérébrale [4, 5]. Les arythmies cardiaques notamment la fibrillation atriale (FA) et le flutter atrial sont retrouvées dans près de 50% d'AVC cardio-emboliques, et la fibrillation atriale non valvulaire (FANV) dans 20-25% de tous les AVC [6-10]. Plusieurs méthodes sont utilisées pour la détection de la FA, parmi lesquelles l'électrocardiogramme standard, le Holter ECG et le monitoring continu, chacune d'elle ayant une sensibilité et une spécificité variables pour la FA paroxystique [11-14]. Afin d'améliorer la prise en charge des patients victimes d'AVC au Congo, nous avons réalisé cette étude avec pour objectifs, d'une part de déterminer la prévalence des troubles rythmiques au cours des infarctus cérébraux et d'autre part, d'identifier les facteurs prédictifs de la FA paroxystique.

## Méthodes

Il s'est agi d'une étude transversale à recueil de données prospectif, descriptive et analytique, réalisée à Brazzaville entre janvier 2012 et décembre 2016. Elle a inclus une série consécutive de patients victimes d'un infarctus cérébral documenté par une tomodensitométrie (TDM) cérébrale ou une imagerie par résonnance magnétique (IRM) et ayant bénéficié d'un Holter ECG des 24 heures dans le cadre de la recherche étiologique. L'échantillon était constitué de 267 patients, répartis en accident ischémique constitué (n = 250), et accident ischémique transitoire (n = 17). Le matériel utilisé comportait un enregistreur CardioMem^®^ CM 3000 à 3 canaux et l'analyse de l'enregistrement était faite à base du logiciel CardioDay^®^ 2.2.0 de GE Health Care, mis en service en août 2009. Les données sociodémographiques et les paramètres cliniques des patients ont été recueillis et analysés. Ainsi, plusieurs variables ont été étudiées, notamment: l'âge; le sexe; le type d'accident vasculaire cérébral ischémique (constitué ou transitoire); les facteurs de risque cardiovasculaire classiques (HTA, diabète, tabagisme); la nature du médecin prescripteur (neurologue, cardiologue, généraliste); les anomalies enregistrées au Holter ECG dès 24h. Les données ont été saisies et analysées avec le logiciel Epi-Info 3.5.3. Les tests de Khi-2 et ANOVA ont permis la comparaison des variables qualitatives et quantitatives. La recherche des facteurs prédictifs de la FA paroxystique s'est faite à l'aide d'une régression logistique. Le seuil de significativité a été fixé à p < 0,05.

## Résultats

**Figure 1:**
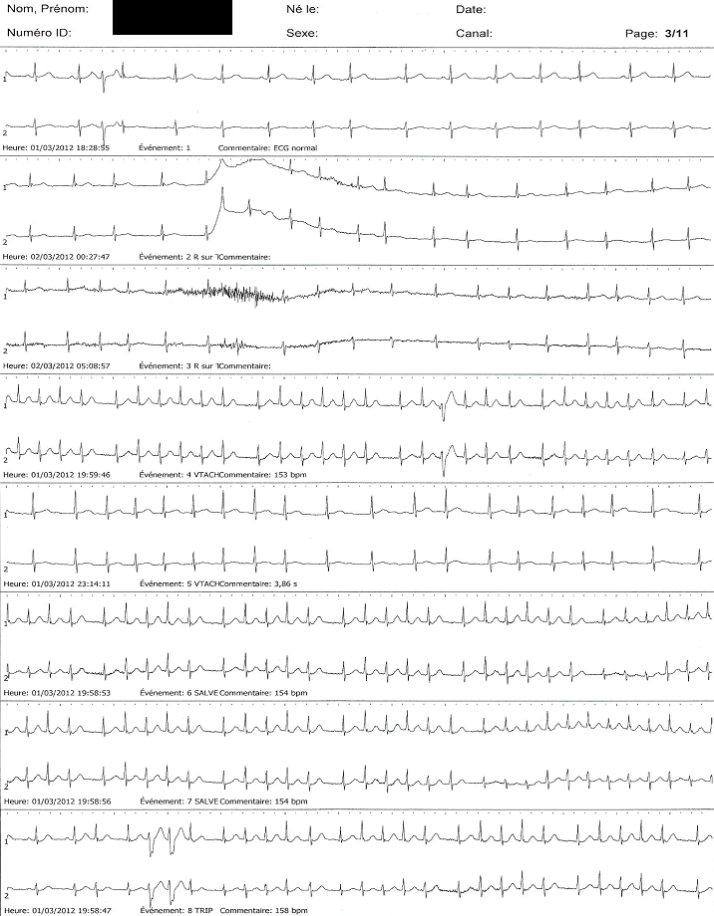
Tracé de fibrillation atriale paroxystique chez un patient de 78 ans, ayant présenté une récidive d’infarctus cérébral

Sur 1075 procédures réalisées, les infarctus cérébraux représentaient la deuxième indication du Holter ECG (n = 267 soit 24,8%), après les palpitations (n = 530 soit 49,3%). Les 267 patients se répartissaient en 164 hommes (61,4%) et 103 femmes (38,6%), âgés en moyenne de 60,2 ± 12,1 ans (extrêmes: 22 et 94 ans), sans difference entre les deux sexes (60,4 ± 10,6 vs. 60,0 ± 14,3 ans; p = 0,826). Les patients âgés de moins de 50 ans représentaient 62 cas (23,4%). Les principaux facteurs de risque cardiovasculaire identifiés étaient une hypertension artérielle (HTA) dans 214 cas (80,1%), un diabète sucré dans 36 cas (13,5%) et un tabagisme dans 18 cas (6,7%), avec un taux de cumul de 1,5 facteur/individu. Les principales caractéristiques des patients sont consignées dans le Tableau 1. L'examen était prescrit par un neurologue dans 137 cas (51,3%), un cardiologue dans 95 cas (35,6%) et un médecin généraliste dans 35 cas (13,1%). Le Holter ECG, normal dans 216 cas (81%), et anormal dans 51 cas (19%), objectivait comme principales anomalies des extrasystoles ventriculaires (ESV) dans 32 cas (12%), une fibrillation atriale (FA) paroxystique dans sept cas (2,6%), des extrasystoles supraventriculaires (ESSV) dans cinq cas (1,8%), une tachycardie ventriculaire (TV) non soutenue dans quatre cas (1,5%), une TV soutenue dans deux cas (0,7%) et un bloc auriculo-ventriculaire (BAV) Mobitz II dans un cas (0,4%). La Figure 1 illustre un tracé de FA paroxystique. En analyse bivariée, il n'a pas été noté de lien entre la FA paroxystique et le sexe (p = 0,55), l'HTA (p = 0,42), le diabète (p = 0,64), et le tabagisme (p = 0,61) En analyse multivariée, seul l'âge (OR = 1,11; p = 0,0134), était considéré comme facteur prédictif de survenue de la FA paroxystique. Le Tableau 2 donne les résultats de la régression logistique.

**Tableau 1:** Caractéristiques des patients

	Patients (N = 267)
Hommes, n (%)	164 (61,4)
Age moyen (ans)	60,2 ± 12,1 (22-94)
Patients < 50 ans, n (%)	62 (23,2)
Accident ischémique transitoire, n (%)	17 (6,4)
Accident vasculaire cérébral constitué, n (%)	250 (93,6)
Hypertension artérielle, n (%)	214 (80,1)
Diabète sucré, n (%)	36 (13,5%)
Tabagisme, n (%)	18 (6,7)
Holter ECG anormal, n (%)	51 (19)
Fibrillation atriale paroxystique, n (%)	7 (2,6)

**Tableau 2:** Régression logistique de la FA paroxystique

Variables explicatives	OR (IC à 95%)	p
Age (ans)	1,11 (1,02-1,20)	0,0134
Diabète sucré (oui/non)	0,79 (0,08-7,49)	0,8388
Hypertension artérielle (oui/non)	1,24 (0,19-7,89)	0,8181
Sexe (Homme/Femme)	1,11 (0,22-5,65)	0,8998
Tabagisme (oui/non)	-	0,9696

## Discussion

Dans notre série, les patients victimes d'infarctus cérébraux sont relativement jeunes, avec un âge moyen autour de 60 ans et une proportion non négligeable des patients de moins de 50 ans, loin de ce qui est rapporté dans les séries occidentales, où les patients sont souvent plus âgés, autour de 66 ans en moyenne [15]. L'hypertension artérielle (HTA), est le facteur de risque cardiovasculaire le plus fréquemment retrouvé, présent chez près de huit patients sur dix dans notre série; constat relevé dans la plupart des séries en Afrique Subsaharienne [16, 17], ce qui en fait le principal facteur de risque d'accidents vasculaires cérébraux ischémiques dans cette partie du continent. La fibrillation atriale (FA) paroxystique, trouble du rythme à l'origine des accidents cardio-emboliques, est relativement peu fréquente dans notre série, retrouvée dans moins de 3% des cas. Dans la littérature, la fréquence de la FA paroxystique au cours des infarctus cérébraux est très variable suivant les séries, allant de 2 à 26% selon la technique d'enregistrement utilisée [13, 14, 18-25], avec une corrélation nette entre la longueur de la durée de l'enregistrement et la fréquence de détection de la FA paroxystique. En effet, dans ces différentes séries, les fréquences de FA les plus faibles ont été obtenues avec les enregistrements durant de 24 à 48 heures [13, 14, 18, 19], alors que les fréquences de FA les plus élevées l'ont été avec des dispositifs implantables, réalisant des enregistrements de longue durée, jusqu'à 1500 heures d'enregistrement en moyenne [20-25]. Il a par ailleurs été démontré un lien entre le nombre d'infarctus cérébraux chez un même sujet et la prépondérance de la FA paroxystique [12], témoignant du rôle majeur de cette arythmie dans les infarctus cérébraux récidivants. Par ailleurs, des études plus récentes ont mis en exergue l'importance de certaines méthodes d'enregistrement dans la détection de la FA paroxystique, avec une sensibilité et une spécificité bien meilleures que celles des méthodes d'enregistrement traditionnelles. C'est le cas du monitoring continu en Unité de Soins Intensifs Neuro-vasculaires (UNV), par méthode conventionnelle ou automatisée [11]; cette dernière rendant possible la détection de la FA paroxystique avec une forte sensibilité dès les 72 premières heures suivant l'admission en UNV. Dans notre étude, concernant la recherche des facteurs prédictifs de survenue de la FA paroxystique au cours des infarctus cérébraux, seul l'âge a été identifié. Dans la littérature, ce constat a également été relevé, la FA paroxystique étant plus fréquente chez les sujets âgés [11, 15], sa fréquence augmentant de façon proportionnelle avec l'âge [11]. Ce constat dénote du fait que, en Afrique Subsaharienne en général, et plus particulièrement au Congo, l'HTA reste le principal facteur étiologique des accidents vasculaires cérébraux [16, 17], survenant chez des sujets relativement jeunes et que la FA paroxystique est l'apanage des sujets âgés, comme cela apparaît dans les séries occidentales.

## Conclusion

Cette étude préliminaire a montré que les troubles du rythme emboligène, au premier rang desquels la fibrillation atriale, sont relativement rares au cours des infarctus cérébraux à Brazzaville. Leur prévalence est probablement sous-estimée, du fait des limites des méthodes d'enregistrement traditionnelles telles que le Holter ECG de 24h, à reconsidérer au profit du monitoring continu en unité de soins intensifs neuro-vasculaires. La fibrillation atriale paroxystique, bien que peu fréquente, reste corrélée à l'âge, principal facteur prédictif. D'où la nécessité de sa recherche systématique chez des sujets âgés, afin de contribuer à améliorer la morbi-mortalité dans cette population vulnérable.

### Etat des connaissances actuelle sur le sujet

- Les accidents cérébro-vasculaires constituent une pathologie fréquente dans le monde, et plus particulièrement en Afrique Sub-saharienne; ils sont l'apanage des sujets âgés en occident;
- L'athérothrombose carotidienne et les causes emboliques, en rapport avec les troubles du rythme cardiaque en sont les principales causes, justifiant le bilan étiologique à visée cardiovasculaire;
- Leur pronostic a été amélioré au cours de ces dernières décennies grâce à la mise en place des unités neurovasculaires, permettant une prise en charge précoce et efficace des patients.

### Contribution de notre étude à la connaissance

- La pathologie intéresse des sujets relativement jeunes dans notre contexte africain, avec une prépondérance de l'hypertension artérielle;
- Les troubles du rythme emboligène, tels que la fibrillation atriale paroxystique sont relativement rares, fréquence probablement sous-estimée en raison des limites de la méthode d'enregistrement utilisée conventionnellement pour leur détection, notamment du fait de la courte durée d'enregistrement de 24h; l'âge est le seul facteur prédictif de survenue de cette arythmie;
- Enfin, le recours à d'autres méthodes de détection plus sensibles et plus spécifiques telles que le monitoring continu en unité de soins intensifs neurovasculaires qui apparaît comme une nécessité, mais cette dernière est peu accessible dans nos conditions d'exercice.

## Conflits d’intérêts

Les auteurs ne déclarent aucun conflit d'intérêts.
